# Supplementary material for: Prevalence of Polymorphism and Post-Training Expression of ACTN3 (R/X) and ACE (I/D) Genes in CrossFit Athletes
Source: Int J Environ Res Public Health. 2023 Mar 1;20(5):4404. doi: 10.3390/ijerph20054404 (PMC10001917; doi:10.3390/ijerph20054404)
Supplement: Supplementary file 1 [file ijerph-20-04404-s001.zip › ijerph-2213866-supplementary.pdf]

En las tablas S se muestran las correlaciones entre las mismas capacidades físicas previas y posteriores al entrenamiento diferenciadas por genotipo.

En la tabla S1. se muestran las correlaciones con el genotipo ACTN3 RR en donde el aumento de la fuerza de los músculos flexores del brazo derecho está directamente relacionado con el aumento de la mayoría de las capacidades físicas evaluadas, donde podemos ver que la potencia de pesos medios y altos estuvieron principalmente relacionadas con la mayoría de las capacidades físicas evaluadas.

Tabla S1.

Correlaciones entre las mismas capacidades físicas por genotipo ACTN3 RR

Variable	Dinamo der (N)	Dinamo izq (N)	RM Front SQ (lb)	RM Shoulder P (lb)	PPower35 (kg)	PPower55 (kg)	PPower75 (kg)	PSplitj35 (kg)	PSplitj55 (kg)	PSplitj75 (kg)
Dinamo der (N)		0.969	0.992		0.970	0.966	0.978	0.972	0.998	0.995
Dinamo izq (N)	0.969			0.980					0.954	0.951
RM Front SQ (lb)	0.992				0.981	0.974	0.973	0.978	0.998	0.999
RM Shoulder P (lb)		0.980								
PPower35 (kg)	0.970		0.981			0.999	0.992	0.999	0.981	0.980
PPower55 (kg)	0.966		0.974		0.999		0.995	1.000	0.977	0.974

PPower75 (kg)	0.978		0.973		0.992	0.995		0.996	0.982	0.976
PSplitj35 (kg)	0.972		0.978		0.999	1.000	0.996		0.981	0.978
PSplitj55 (kg)	0.998	0.954	0.998		0.981	0.977	0.982	0.981		0.999
PSplitj75 (kg)	0.995	0.951	0.999		0.980	0.974	0.976	0.978	0.999	

Dinamo der= Dinamometría derecha; Dinamo izq= Dinamometía izquierda; ClasDinamo der= Clasificación dinamometría derecha; ClasDinamo izq= Clasificación dinamometría izquierda; RM Front SQ= Repetición máxima de front squat; RM Shoulder P= Repetición máxima de shoulder press; VO2max= Consumo máximo de oxígeno; ClasVO2max= Clasificación de consumo máximo de oxígeno; PPower35= Potencia en power clean a 35%; PPower55= Potencia en power clean a 55%; PPower75= Potencia en power clean a 75%; PSplit35= Potencia en Split jerk a 35%; PSplit55= Potencia en Split jerk a 55%; PSplit75= Potencia en Split jerk a 75%.

En la tabla S2. se muestran las correlaciones con el genotipo ACTN3 RX en donde el aumento de la fuerza máxima de Front Squat está principalmente relacionado con el aumento de la mayoría de las capacidades físicas evaluadas, de la misma manera, la potencia con pesos altos estuvo principalmente relacionada con la mayoría de las capacidades físicas evaluadas. Entre los diferentes genotipos evaluados, fue el único que relaciono el aumento de la fuerza máxima de Front Squat con el consumo máximo de oxígeno.

Tabla S2.

Correlaciones entre las mismas capacidades físicas por genotipo ACTN3 RX

Variable	Dinamo der (N)	Dinamo izq (N)	RM Front SQ (lb)	RM Shoulder P (lb)	VO^2max (ml/kg/min)	PPower35 (kg)	PPower55 (kg)	PPower75 (kg)	PSplitj35 (kg)	PSplitj55 (kg)	PSplitj75 (kg)
Dinamo der (N)		0.962	0.875	0.925		0.742	0.747	0.715			0.898

Dinamo izq (N)	0.962		0.800	0.947		0.790	0.708				0.901
RM Front SQ (lb)	0.875	0.800		0.875	0.767	0.737	0.801	0.783			0.717
RM Shoulder P (lb)	0.925	0.947	0.875			0.711					0.761
VO^2max (ml/kg/min)			0.767								
PPower35 (kg)	0.742	0.790	0.737	0.711			0.909	0.699			0.818
PPower55 (kg)	0.747	0.708	0.801			0.909		0.901	0.680	0.822	0.853
PPower75 (kg)	0.715		0.783			0.699	0.901			0.686	0.809
PSplitj35 (kg)							0.680			0.898	
PSplitj55 (kg)							0.822	0.686	0.898		
PSplitj75 (kg)	0.898	0.901	0.717	0.761		0.818	0.853	0.809			

Dinamo der= Dinamometría derecha; Dinamo izq= Dinamometía izquierda; ClasDinamo der= Clasificación dinamometría derecha; ClasDinamo izq= Clasificación dinamometría izquierda; RM Front SQ= Repetición máxima de front squat; RM Shoulder P= Repetición máxima de shoulder press; VO2max= Consumo máximo de oxígeno; ClasVO2max= Clasificación de consumo máximo de oxígeno; PPower35= Potencia en power clean a 35%; PPower55= Potencia en power clean a 55%; PPower75= Potencia en power clean a 75%; PSplit35= Potencia en Split jerk a 35%; PSplit55= Potencia en Split jerk a 55%; PSplit75= Potencia en Split jerk a 75%.

En la tabla S3. se muestran las correlaciones con el genotipo ACTN3 XX en donde el aumento de la fuerza máxima de Shoulder Press está principalmente relacionado con el aumento de la mayoría de las capacidades físicas evaluadas, así mismo, la potencia con pesos bajos estuvo directamente relacionada con la mayoría de las capacidades físicas evaluadas.

Tabla S3.

Correlaciones entre las mismas capacidades físicas por genotipo ACTN3 XX

Variable	Dinamo der (N)	Dinamo izq (N)	RM Front SQ (lb)	RM Shoulder P (lb)	VO^2max (ml/kg/min)	PPower35 (kg)	PPower55 (kg)	PPower75 (kg)	PSplitj35 (kg)	PSplitj55 (kg)	PSplitj75 (kg)
Dinamo der (N)		0.937		0.914		0.885	0.879				
Dinamo izq (N)	0.937		0.917	0.935		0.932	0.931			0.915	
RM Front SQ (lb)		0.917		0.961		0.939			0.986	0.998	0.989
RM Shoulder P (lb)	0.914	0.935	0.961			0.950	0.881		0.958	0.964	0.955
VO^2max (ml/kg/min)											
PPower35 (kg)	0.885	0.932	0.939	0.950			0.978	0.979	0.933	0.958	0.951
PPower55 (kg)	0.879	0.931		0.881		0.978		0.987		0.895	
PPower75 (kg)						0.979	0.987			0.902	0.898
PSplitj35 (kg)		0.915	0.998	0.964		0.958	0.895	0.902	0.990		0.955
PSplitj55 (kg)			0.986	0.958		0.933				0.990	0.997

PSplitj75 (kg)			0.989	0.955		0.951		0.898	0.997	0.995	

Dinamo der= Dinamometría derecha; Dinamo izq= Dinamometía izquierda; ClasDinamo der= Clasificación dinamometría derecha; ClasDinamo izq= Clasificación dinamometría izquierda; RM Front SQ= Repetición máxima de front squat; RM Shoulder P= Repetición máxima de shoulder press; VO2max= Consumo máximo de oxígeno; ClasVO2max= Clasificación de consumo máximo de oxígeno; PPower35= Potencia en power clean a 35%; PPower55= Potencia en power clean a 55%; PPower75= Potencia en power clean a 75%; PSplit35= Potencia en Split jerk a 35%; PSplit55= Potencia en Split jerk a 55%; PSplit75= Potencia en Split jerk a 75%.

En la tabla S4. se muestran las correlaciones con el genotipo ACE DD en donde el aumento de la fuerza de los músculos flexores del brazo derecho está directamente relacionado con el aumento de la mayoría de las capacidades físicas evaluadas; de la misma manera la potencia con pesos bajos y moderados estuvo principalmente relacionada con la mayoría de las capacidades físicas evaluadas.

Tabla S4.

Correlaciones entre las mismas capacidades físicas por genotipo ACE DD

Variable	Dinamo der (N)	Dinamo izq (N)	RM Front SQ (lb)	RM Shoulder P (lb)	VO^{2}max (ml/kg/min)	PPower35 (kg)	PPower55 (kg)	PPower75 (kg)	PSplitj35 (kg)	PSplitj55 (kg)	PSplitj75 (kg)
Dinamo der (N)		0.977	0.988	0.952		0.975			0.992	0.982	0.967
Dinamo izq (N)	0.977		0.990	0.995		0.955			0.955		
RM Front SQ (lb)	0.988	0.990		0.980		0.987			0.983	0.955	
RM Shoulder P (lb)	0.952	0.995	0.980								
VO^{2}max (ml/kg/min)											

PPower35 (kg)	0.975	0.955	0.987				0.959		0.990	0.970	0.958
PPower55 (kg)						0.959		0.999	0.957	0.962	0.969
PPower75 (kg)							0.999			0.953	0.962
PSplitj35 (kg)	0.992	0.955	0.983			0.990	0.957			0.993	0.984
PSplitj55 (kg)	0.982		0.955			0.970	0.962	0.953	0.993		0.998
PSplitj75 (kg)	0.967					0.958	0.969	0.962	0.984	0.998	

Dinamo der= Dinamometría derecha; Dinamo izq= Dinamometía izquierda; ClasDinamo der= Clasificación dinamometría derecha; ClasDinamo izq= Clasificación dinamometría izquierda; RM Front SQ= Repetición máxima de front squat; RM Shoulder P= Repetición máxima de shoulder press; VO2max= Consumo máximo de oxígeno; ClasVO2max= Clasificación de consumo máximo de oxígeno; PPower35= Potencia en power clean a 35%; PPower55= Potencia en power clean a 55%; PPower75= Potencia en power clean a 75%; PSplit35= Potencia en Split jerk a 35%; PSplit55= Potencia en Split jerk a 55%; PSplit75= Potencia en Split jerk a 75%.

En la tabla S5. se muestran las correlaciones con el genotipo ACE ID en donde el aumento de la fuerza máxima de Front Squat está directamente relacionado con el aumento de la mayoría de las capacidades físicas evaluadas, de la misma manera la potencia con pesos bajos, la cual estuvo directamente relacionada con la mayoría de las capacidades físicas evaluadas.

Tabla S5.

Correlaciones entre las mismas capacidades físicas por genotipo ACE ID

Variable	Dinamo der (N)	Dinamo izq (N)	RM Front SQ (lb)	RM Shoulder P (lb)	VO^2max (ml/kg/min)	PPower35 (kg)	PPower55 (kg)	PPower75 (kg)	PSplitj35 (kg)	PSplitj55 (kg)	PSplitj75 (kg)
Dinamo der (N)		0.942	0.804	0.922		0.609		0.662			0.842
Dinamo izq (N)	0.942		0.811	0.934		0.657					0.904
RM Front SQ (lb)	0.804	0.811		0.885		0.761	0.751	0.797	0.756	0.826	0.839
RM Shoulder P (lb)	0.922	0.934	0.885					0.681			0.854
VO^2max (ml/kg/min)											
PPower35 (kg)	0.609	0.657	0.761				0.910	0.690	0.825	0.809	0.686
PPower55 (kg)			0.751			0.910		0.836	0.766	0.730	
PPower75 (kg)	0.662		0.797	0.681		0.690	0.836				
PSplitj35 (kg)			0.756			0.825	0.766			0.964	
PSplitj55 (kg)			0.826			0.809	0.730		0.964		0.644

PSplitj75 (kg)	0.842	0.904	0.839	0.854		0.686				0.644	

Dinamo der= Dinamometría derecha; Dinamo izq= Dinamometía izquierda; ClasDinamo der= Clasificación dinamometría derecha; ClasDinamo izq= Clasificación dinamometría izquierda; RM Front SQ= Repetición máxima de front squat; RM Shoulder P= Repetición máxima de shoulder press; VO2max= Consumo máximo de oxígeno; ClasVO2max= Clasificación de consumo máximo de oxígeno; PPower35= Potencia en power clean a 35%; PPower55= Potencia en power clean a 55%; PPower75= Potencia en power clean a 75%; PSplit35= Potencia en Split jerk a 35%; PSplit55= Potencia en Split jerk a 55%; PSplit75= Potencia en Split jerk a 75%.

En la tabla S6. se muestran las correlaciones con el genotipo ACE II en donde relaciono únicamente el aumento de la fuerza máxima de los músculos flexores del brazo izquierdo con el aumento en la potencia con pesos bajos. Por otra parte, el aumento en la fuerza máxima de Shoulder Press disminuyo la potencia con pesos medios. El aumento en la potencia con pesos medios disminuye la potencia con pesos altos.

Tabla S6.

Correlaciones entre las mismas capacidades físicas por genotipo ACE II

Variable	Dinamo der (N)	Dinamo izq (N)	RM Front SQ (lb)	RM Shoulder P (lb)	VO^2max (ml/kg/min)	PPower35 (kg)	PPower55 (kg)	PPower75 (kg)	PSplitj35 (kg)	PSplitj55 (kg)	PSplitj75 (kg)
Dinamo der (N)											
Dinamo izq (N)						0.998					
RM Front SQ (lb)											
RM Shoulder P (lb)							-0.997				

VO^{2}max (ml/kg/min)				
PPower35 (kg)	0.998			
PPower55 (kg)		-0.997		-1.000
PPower75 (kg)			-1.000	
PSplitj35 (kg)				
PSplitj55 (kg)				
PSplitj75 (kg)				

Dinamo der= Dinamometría derecha; Dinamo izq= Dinamometía izquierda; ClasDinamo der= Clasificación dinamometría derecha; ClasDinamo izq= Clasificación dinamometría izquierda; RM Front SQ= Repetición máxima de front squat; RM Shoulder P= Repetición máxima de shoulder press; VO2max= Consumo máximo de oxígeno; ClasVO2max= Clasificación de consumo máximo de oxígeno; PPower35= Potencia en power clean a 35%; PPower55= Potencia en power clean a 55%; PPower75= Potencia en power clean a 75%; PSplit35= Potencia en Split jerk a 35%; PSplit55= Potencia en Split jerk a 55%; PSplit75= Potencia en Split jerk a 75%.
